# Room of horrors intervention in the nursing home sector – a German pre-post-follow-up longitudinal study

**DOI:** 10.1186/s12913-025-13823-1

**Published:** 2025-11-27

**Authors:** Johannes Gräske, Graciana-Virginye Versteeg, Theresa A. Forbrig

**Affiliations:** 1 Department II: Health, Education, and Social Sciences, Alice Salomon University of Applied Science, Alice-Salomon-Platz 5, 12627 Berlin, Germany; 2 Pflegewohnzentrum Kaulsdorf-Nord gGmbH, Tangermünder Str. 30, 12627 Berlin, Germany; 3 Charité – Universitätsmedizin Berlin, Charitéplatz 1, 10117 Berlin, Germany

**Keywords:** Patient safety, Nursing homes, Simulation training, Error reporting

## Abstract

**Background:**

Patient safety is a significant aspect of healthcare delivery. However, research on interventions for improving patient safety culture in nursing homes is scarce. This study evaluated the effectiveness of a low-fidelity Room of Horrors (RoH) simulation on the attitudes of nursing home staff toward patient safety with a three-month follow-up.

**Methods:**

This pre-post-follow-up longitudinal interventional study encompassed five nursing homes in Berlin, Germany. An RoH training session, including error detection and structured debriefing, was offered to all the nursing staff. Attitudes toward patient safety were assessed before, immediately after, and three months after the intervention using the German short version of the Attitudes to Patient Safety Questionnaire (G-APSQ-short). Generalized linear models (GLMs) were used to analyze the data for time effects and the influence of professional experience and group size.

**Results:**

A total of 123 participants completed the baseline survey, and 66 completed the three-month follow-up. There were minimal changes in the nursing staff’s attitude toward patient safety. However, modest correlations were observed between professional experience and changes in attitude and between group size and changes in attitude. Smaller groups had higher post-intervention scores, with the effect attenuating over time.

**Conclusions:**

RoH simulations represent a feasible strategy for enhancing patient safety awareness among nursing home staff. However, sustained attitudinal change may require follow-up interventions and integration into more far-reaching patient safety programs.

**Clinical trial number:**

Not applicable.

## Background

Due to an increased number of people utilizing healthcare services worldwide, patient safety has become an important aspect of healthcare delivery. The WHO has formulated the following characteristics: Patient safety should be understood as a framework of organized activities that take into account, among other things, processes, procedures, behaviors, technologies, and environments in healthcare that “consistently and sustainably lower risks, reduce the occurrence of preventable harm, make errors less likely, and reduce their impact where they do occur” [1]. Around one in ten patients is harmed in the course of healthcare, with more than three million people dying from these consequences every year [2]. 

### Causes of patient safety failures

Most failures (more than 50%) are due to medication management [3, 4]. The error categories with particular frequency, besides medication errors, are unsafe surgical procedures, infections, diagnostic errors, patient falls, pressure ulcers, incorrect identification of patients, unsafe blood transfusions, and venous thromboembolism [1].

### Patient safety in nursing homes

Nursing homes have become a major living arrangement for older adults with complex nursing care needs. A qualitative systematic review included 12 studies investigating the barriers and facilitators of patient safety in residential settings and nursing homes [5]. The results showed that material resources, management, communication, the optimal use of expertise, and the effective use of guidelines are essential for patient safety [5].

Such risks can arise due to the special requirements of the intended home setting. In addition, the complex to highly complex care needs and the associated vulnerability of the residents increase the safety challenges. Long-term care facilities also often offer a broader range of care services, which requires equally broad expertise on the part of the caregivers. At the same time, there is a balance between risk prevention and an individual’s concept of quality of life [6], such as the desire for independent mobility despite fall risks or maintaining personal routines. Qualitative research has also shown that quality in nursing homes is understood not only as the prevention of harm but as a relational concept shaped by professional competence, dignity, and continuity of care [7], which supports initiatives to strengthen evidence-based assurance of resident safety [5]. A systematic review by Keers et al. indicated that drug administration errors in healthcare institutions are due mainly to lapses in drug knowledge and willful non-compliance with the guidelines [8]. A similar assumption was made for the 40% of hospital admissions from long-term care facilities [9].

### Enhancing patient safety

According to research, errors in care are preventable in more than half the cases. Mistakes and risks, such as medication errors and their causes, are multifactorial, which technology alone cannot solve [10]. Therefore, staff characteristics, particularly attitudes toward patient safety, have become a focus for improvement. Attitudes toward patient safety can be defined as the human beliefs and behaviors that can affect decisions and influence behavior [11]. Determining nurses’ attitudes toward patient safety helps to identify targeted educational and organizational interventions that address negative beliefs and support positive safety behaviors [12]. The study findings of [12] revealed less-than-positive safety attitudes across all safety domains among both nurses and doctors. While nurses reported lower scores in teamwork climate, doctors rated work conditions more negatively, reflecting differing safety concerns between the professional groups. Education in safety is associated with better patient outcomes [13]. Comprehensive patient safety education programs, using a combination of lectures, discussions, and simulation, have been shown to improve nurses’ safety knowledge, attitudes, and skills [14]. However, outcome evaluations still rarely assess long-term behavioral change or safety culture developme [14]. Despite the recognized importance of patient safety education, studies indicate that existing nursing curricula often address safety in a fragmented way and do not consistently integrate system-level safety concepts or human factors [15]. Simulation-based learning, such as the Room of Horrors (RoH), addresses this gap by providing a realistic, interactive environment where healthcare professionals can actively engage in identifying and analyzing safety hazards. However, implementing such education formats can be difficult in busy and short-staffed teams. Additionally, changing individual, professional, and cultural attitudes toward safety remains a challenge, despite educational efforts.

One approach that aims to address these challenges is the Room of Horrors (RoH). The RoH concept is a low-threshold and cost-effective training method for employees in long-term care facilities and is classified as a low-fidelity simulation. The concept involves simulating real care situations with hidden patient safety risks and errors that healthcare professionals should detect and reflect upon [16]. In a study by Wiest et al. (2017), 125 female doctors took part in an RoH scenario, and 120 completed a follow-up survey one month later. The result was that they were more attentive to the dangers in patient care due to the simulation. In addition, 52.1% of the participants took action in connection with a potential source of danger [17]. Evaluations from Switzerland also showed that the RoH concept is an effective method that could heighten employees’ situational awareness of the safety risks and was rated by the participants as recommendable, relevant, and instructive [16, 18]. Daupin et al. (2016) showed that most participants (114/136, 84.4% [sic]) intended to change their practices because of their exposure to the simulation [10].

Lee et al. (2023) also concluded that RoH simulation is a valuable teaching tool for healthcare students and professionals, although research is still needed on, for example, optimal group size [19].

With regard to long-term care, little data is available on the effectiveness of the RoH concept, particularly concerning attitudes toward errors, risks, and the potential dangers in long-term care. These findings highlight the urgent need to enhance patient safety practices in nursing homes. This study aimed to evaluate the impact of a low-fidelity Room of Horrors (RoH) intervention on the attitude toward patient safety among nursing home staff in Berlin, Germany.

## Methods

A pre-post-follow-up longitudinal interventional (6/2024–07/2024) study was conducted within *n* = 5 nursing homes and a follow-up after three months (9/2024–10/2024). All nursing homes belonged to the same provider and were based in Berlin/Germany.

### Sample

The study was designed as a complete survey and included all the nurses (registered and assistant) from the participating nursing homes. All the nurses were invited to participate and were given a dedicated time slot in the roster. However, participation was voluntary. All participants had to be at least 18 years old. Exclusion criteria for the study were being a nursing student or refusing to participate.

### Intervention

The intervention consisted of a Room of Horrors (RoH) simulation, specifically designed for the nursing home setting. It followed a three-step structure consisting of pre-briefing, intervention, and debriefing. The scenario was collaboratively developed by six practical instructors from the participating nursing homes to reflect real-life safety risks in long-term care, following the WHO’s International Classification for Patient Safety [20]. Error types that are not applicable to the nursing home context, such as transfusion-related incidents, were deliberately excluded. The final error categories included and error descriptions are displayed in the Table 1:

**Table 1:** Built-in errors in the room of horrors simulation

Error category (20)	Error description
Medical device	• Wheelchair: brake not functional
	• Walker: uneven handle height, brake not functional
Resident accidents/ Infrastructure	• Night lamp: bulb defective
Nutrition management	• Chicken egg present despite egg white allergy
Medication management	• Metamizole allergy documented + Novalgin prescribed
	• Novaminsulfone drops prepared in advance
Clinical process	• Incorrect wound dressing material
	• Incontinence material twisted, front/back reversed
Nosocomial infection	• NaCl 0.9% without date of opening and with cannula inserted
	• Coffee cup covered with mold
Documentation	• Wound documentation missing
Clinical process	• Compression stockings wrinkled, one turned inside out
	• Bed rail raised
	• Cannula cap under resident

In total, *n* = 14 errors were included in the RoH. The number was a typical number of errors in RoH training [19]. To reflect everyday work settings and team dynamics, participants completed the scenario in existing ward teams (“real groups”), composed of 3 to 8 people. This setup supported authentic communication and teamwork during the exercise. In total, *n* = 33 groups participated in the RoH intervention. These groups mirrored existing care teams on the wards and were chosen not only to reflect real-life team dynamics but also to support the logistical integration of the intervention into daily operations.

The RoH was placed in the training center of the nursing home provider. Before entering the RoH, all participants were given a short verbal introduction, emphasizing free exploration of the environment. This included the participants being allowed to touch, remove, and turn everything within the indicated area. Indication was done with marks on the floor. No further instructions (e.g., how to organize) were provided. In the room, the participants found the residents’ records for further information about the nursing situation and a protocol to document the errors they found. Each team participated in the Room of Horrors scenario for a total of 15 min. Afterward, a structured debriefing followed the principles of the 3D method. The modified 3D method [21] steps are diffusing, discovering, and deepening, which empower participants to reflect on their experience, process emotional reactions, and translate insights into future clinical practice. This approach supports structured reflection, encourages emotional processing, and helps participants connect their simulation experience to everyday clinical practice.The debriefing was scheduled for 45 min and was conducted individually with each group. The same instructors who conducted the pre-briefing also performed the debriefing.

### Instrument

A written standardized questionnaire was used. The pre-questionnaire comprised two parts. The first part contained personal (e.g., age, sex) and professional (e.g., working experiences, being a registered nurse — yes/no) questions. An individual pseudonym was created by answering special questions (e.g., what is the third letter of your birth name). This was necessary to connect individual data in the long run. In the second part, we evaluated the attitude toward patient safety with the G-APSQ-short [22]. The questionnaire comprised 14 statements (e.g., human failures are inevitable). Using a 7-point Likert scale, the participants could rate whether they strongly disagreed (1 point) or strongly agreed (7 points). The G-APSQ-short contained six subdomains: patient safety training (3 statements), error reporting (2 statements), working hours (3 statements), error inevitability (2 statements), patient role in the error (2 statements), and the importance of patient safety in the curriculum (2 statements). In addition, a total score, including all 14 items, was calculated. The subdomain and total scores were calculated using the included statements, resulting in a theoretical range of 1–7, with higher scores indicating a better attitude. The G-APSQ-short was originally developed for medical students. However, Kaveh, Sharif-Nia [23] developed a version for nurses with good psychometric properties. Based on these findings, we adapted the G-APSQ-short wording of several items to match the nursing home context. The personal and professional questions were excluded from the post and long questionnaire. Only the G-APSQ-short was completed by the participants. In addition, we asked whether the participants would recommend the RoH to other nurses as a practical training, with answers from 1 (no) to 5 (for sure).

### Data collection

The pre- and post-data collection was conducted immediately before the RoH and directly after the debriefing, respectively. An online survey tool (Quamp^®^^−^ https://www.sociolutions.de/) from the university was used to collect the data. All participants were offered a room with several PCs to complete the questionnaire. Refusing to complete the questionnaire did not lead to exclusion from the RoH. The third data collection (long run) was conducted three months after the RoH intervention. Participants received an email invitation with a link to the online survey and two reminder emails, each sent one week apart.

### Data analysis

First, data were checked for outliers in the Likert Scale responses using descriptive statistics, No outliers were identified, and all data were retained for analysis. No imputation of missing data was performed. Data description was done using typical parameters such as mean and standard deviation (SD). For group comparisons between participants who dropped out and those who completed the three-month follow-up, independent-samples t-tests were applied to continuous variables (age, working experience, time with the same employer), and Chi-square test and Fisher’s exact test was used for categorical variables (group size, sex, profession). These comparisons refer to the baseline characteristics of the two subsamples. Generalized linear models (GLMs) were used to analyze the developments of participant responses over time. Dependent variables were the pre-, post-, and long-run scores of the G-APSQ-short subdomains and the total score. The influencing factors were being an RN (yes/no), group size (≤ 4/>4), and covariate working experience in years. Due to the small number of participants after three months and to increase the stability of the model, the group size was transformed into a bivariate item (≤ 4/>4). Because of the multicollinearity with age and time with the same employer, only working experience (in years) could be included in the GLMs. Model assumptions (e.g., normal distribution, homoscedasticity, and sphericity) were considered for all analyses. The assumption of sphericity was tested and, where violated, the Greenhouse–Geisser correction was applied in the GLM analyses. Table 5 presents the results with these corrections already incorporated. Effect sizes for the GLM analyses are reported as partial eta squared (ηp²p), representing the proportion of variance explained by each factor, accounting for other variables in the model. Model assumptions (e.g., normal distribution or homoscedasticity) were considered for all analyses. Data description and analysis were conducted with SPSS^®^ 29. All tests were interpreted based on a significance level of 0.05.

## Results

In total, 178 people were invited and received the study information, and *n* = 123 people participated in the RoH intervention and data collection. All participants completed the pre- and post-evaluation, while only *n* = 66 participants completed the questionnaire three months after the intervention, resulting in a drop-out rate of 46.3%. The drop-out rate was 46.3%. The drop-out group consisted of 57 participants, which includes those who did not respond at follow-up and six participants whose pseudonyms could not be matched to their baseline data, making their follow-up data unusable for longitudinal analysis. Another main reason for dropping out was not following the email invitation to complete the questionnaire. The long-run participants comprised mainly older (mean 47.0 years), female (86.4%), and registered (56.1%) nurses (see Table 2). However, none of the characteristics showed significant differences between the participants completing the long run and dropping out.

**Table 2:** Characteristics of the participants

	Baseline *n* = 123	Drop-out *n* = 57	Follow-up *n* = 66	*p*-value
**Age** in years, mean (SD)	48.1 (10.7)	49.4 (10.7)	47.0 (10.6)	0.231
**Working experience** in years, mean (SD)	16.2 (11.1)	15.6 (11.6)	16.7 (10.6)	0.594
**Time with the same employer** in years, mean (SD)	11.4 (8.7)	10.7 (8.7)	12.0 (8.7)	0.430
**Sex** n (%)				0.258
Female	110 (89.4)	53 (93.0)	57 (86.4)	
Male	13 (10.6)	4 (7.0)	9 (13.6)	
**Profession** n (%)				0.703
Nursing assistant	56 (45.5)	27 (47.4)	29 (43.9)	
Registered nurse	67 (54.5)	30 (52.6)	37 (56.1)	

*p*-values refer to comparisons between participants who dropped out and those who completed the three-month follow-up. Independent-samples t-tests were used for continuous variables; Fisher’s exact tests were used for categorical variables (sex, profession)

The baseline sample was divided into *n* = 33 groups. None of the groups dropped off in total. Most of the nurses participated in groups of either *n* = 4 or *n* = 5 (39.4 and each). Further information about the intervention is displayed in Table 3.

**Table 3:** Information about the RoH

	Baseline *n* = 123	Drop-out *n* = 57	Follow-up *n* = 63	*p*-value
**Group size** n (%)				0.129
**≤**3	26 (21.1)	15 (26.3)	11 (16.7)	
4	38 (30.9)	12 (21.1)	26 (39.4)	
5	51 (41.5)	25 (43.9)	26 (39.4)	
**≥** 6	8 (6.5)	5 (8.8)	3 (4.5)	
**Number of identified errors**, mean (SD)	8.4 (2.6)	8.0 (2.1)	8.8 (2.8)	0.086
**Recommendation of the RoH**, median	5 (strongly)	5 (strongly)	5 (strongly)	0.051

*p*-values refer to comparisons between participants who dropped out and those who completed the three-month follow-up. Independent-samples t-tests were used for continuous variables with normal distributions; the chi-square test was used for the categorical variable “Group size”; and the Mann–Whitney U test was applied for the ordinal variable “Recommendation of the RoH“

At baseline, all the nurses showed a high attitude toward patient safety. The subdomain that received the lowest mean score was the ‘patient’s role in the error,’ which was 4.9 (1.6), while the highest score was attributed to the ‘importance of patient safety in the curriculum,’ recorded at 6.0 (1.3). This was also the only subdomain where a statistically significant difference between drop-out (5.7 (1.5)) and follow-up (6.2 (1.0)) was found (see Table 4).

**Table 4:** Baseline G-APSQ-short scores

	Baseline *n* = 123	Drop-out *n* = 57	Follow-up *n* = 63	*p*-value
Patient safety training	5.4 (1.4)	5.3 (1.5)	5.4 (1.4)	0.710
Error reporting	5.5 (1.4)	5.4 (1.5)	5.6 (1.3)	0.328
Working hours	5.3 (1.4)	5.1 (1.5)	5.5 (1.4)	0.068
Error inevitability	5.8 (1.3)	5.6 (1.4)	6.0 (1.2)	0.118
Patient’s role in the error	4.9 (1.6)	4.7 (1.7)	5.0 (1.6)	0.368
Importance of patient safety in the curriculum	6.0 (1.3)	5.7 (1.5)	6.2 (1.0)	0.030
Total score	5.4 (1.0)	5.3 (1.1)	5.6 (0.9)	0.097

Theoretical range: 1–7, Values are presented as means, with standard deviations in brackets. *p*-values refer to differences between time points

**Table 5:** The G-APSQ-short scores for the follow-up group

	Pre *n* = 66	Post *n* = 66	Long *n* = 66
Patient safety training	5.4 (1.4)	5.9 (1.1)	5.6 (1.2)
Error reporting	5.6 (1.3)	5.7 (1.2)	5.8 (1.1)
Working hours	5.5 (1.4)	5.8 (1.2)	5.8 (1.2)
Error inevitability	6.0 (1.2)	6.1 (1.1)	6.1 (0.8)
Patient’s role in the error	5.0 (1.6)	4.9 (1.6)	5.1 (1.5)
Importance of patient safety in the curriculum	6.2 (1.0)	6.3 (1.0)	6.3 (0.8)
Total score	5.6 (0.9)	5.8 (0.8)	5.7 (0.7)

Theoretical range: 1–7, Values are presented as means, with standard deviations in brackets. *p*-values refer to differences between time points

Crude changes in the G-APSQ-short scores of all subdomains are shown in Table 5. The subdomains with the least changes over time were ‘error inevitability’ and the ‘importance of patient safety in the curriculum,’ with a maximum change of 0.1 scores between the pre- and post-evaluations.

**Table 6:** Associations with the G-APSQ-short scores over time

	Within-subject effects			Between-subject effects			
		*p*-value	ηp2		(co-)variable	*p*-value	ηp2
Patient safety training	Time	0.171	0.209		**Constant term**	**< 0.001**	**0.919**
	Time*profession	0.302	0.019		Profession	0.995	0.000
	Time*group size	0.242	0.023		Group size	0.216	0.025
	Time*work experience	0.523	0.009		Work experience	0.149	0.034
Error reporting*	Time	0.315	0.018		**Constant term**	**< 0.001**	**0.906**
	Time*profession	0.619	0.007		Profession	0.749	0.002
	Time*group size	0.932	0.001		Group size	0.611	0.004
	Time*work experience	0.466	0.011		Work experience	0.325	0.016
Working hours	Time	0.085	0.042		**Constant term**	**< 0.001**	**0.898**
	Time*profession	0.083	0.043		Profession	0.161	0.032
	Time*group size	0.510	0.010		Group size	0.768	0.001
	Time*work experience	**0.048**	**0.053**		Work experience	0.453	0.009
Error inevitability	Time	0.065	0.046		**Constant term**	**< 0.001**	**0.938**
	Time*profession	0.297	0.020		Profession	0.252	0.021
	Time*group size	0.486	0.011		Group size	0.186	0.028
	Time*work experience	**0.016**	**0.069**		Work experience	0.959	0.000
Patient role in error	Time	0.471	0.012		**Constant term**	**< 0.001**	**0.812**
	Time*profession	0.175	0.029		Profession	0.594	0.005
	Time*group size	0.286	0.020		Group size	0.186	0.029
	Time*work experience	0.365	0.019		Work experience	0.978	0.000
Importance of patient safety in the curriculum	Time	0.837	0.002		**Constant term**	**< 0.001**	**0.942**
	Time*profession	0.381	0.015		Profession	0.292	0.018
	Time*group size	**0.033**	**0.057**		Group size	0.075	0.051
	Time*work experience	0.571	0.009		Work experience	0.211	0.026
Total score	Time	0.261	0.022		**Constant term**	**< 0.001**	**0.953**
	Time*profession	0.334	0.017		Profession	0.334	0.015
	Time*group size	0.483	0.010		Group size	0.266	0.020
	Time*work experience	0.184	0.028		Work experience	0.262	0.021

All: Greenhouse–Geisser, bold values are significant to α = 0.05; ηp2 = partial eta squared

**Fig. 1:**
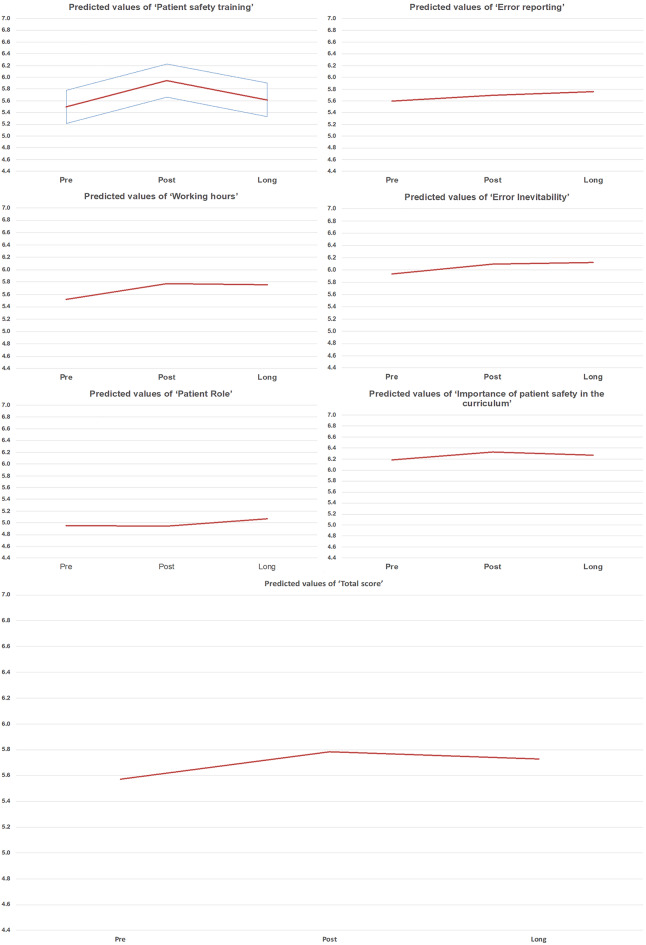
Predicted values of the G-APSQ-short subscale scores across three measurement points (pre, post, follow-up) for each subdomain and the total score. Subfigures 1 and 2 display predicted values by quartiles of work experience; subfigure 3 displays values by group size. The blue shaded areas represent 95% confidence intervals of the predicted values. Theoretical score range for all subdomains and the total score is 1 to 7, with higher scores indicating a more positive attitude toward patient safety

**Fig. 2:**
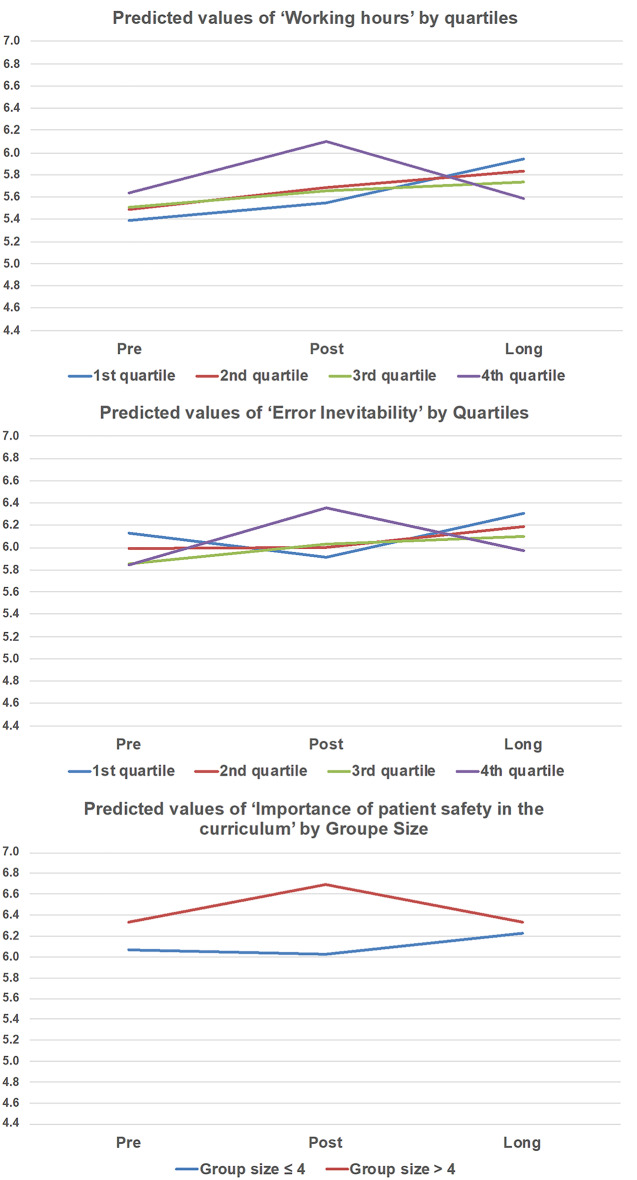
The predicted values of the G-APSQ-short scores by quartiles and Group size Theoretical range: 1–7

Associations of the within-subject effects in the G-APSQ-short scores (see Table 6) were found for time in combination with working experience in the subdomains of ‘working hours’ (*p* = 0.048) and ‘error inevitability’ (*p* = 0.016). In addition, an effect for the combination of time and group size was found for the subdomain ‘importance of patient safety in the curriculum’ (*p* = 0.033). No other effects were found either within or between subjects. Figures 1 and 2 present exploratory visualizations of predicted G-APSQ-short values over time by subdomain, work experience, and group size. While no statistically significant differences were found, the plots illustrate consistent trends across several subgroups that may inform future hypothesis development. Figure 1 presents the predicted values and confidence intervals for all G-APSQ-short subdomains and the total score. These analyses are exploratory and intended to visualize possible trends in the data. Figure 2 presents exploratory subgroup analyses, showing predicted values for the subdomains that demonstrated significant interaction effects over time with baseline quartiles or group size. While the study was not designed for confirmatory subgroup analysis, these visualizations help to illustrate potential differences in safety attitudes between participant groups. For ‘working hours’ and ‘error inevitability,’ the nurses with a longer work experience (4. quartile) showed increased values in the post-evaluation with a decline at the long (three-month) evaluation. All the others showed slightly increased values. Regarding the group size, the predicted values for the subdomain “importance of patient safety in the curriculum” were higher in the post-evaluation for the larger groups (> 4 participants) compared to the smaller groups (≤ 4 participants). However, after three months, both group sizes had converged.

## Discussion

This study evaluated the association between a low-fidelity RoH intervention and attitudes toward patient safety among nursing home staff. The findings indicated only a small, statistically non-significant changes in patient safety attitudes after three months. The GLM analysis showed a minor association between changes in attitude toward patient safety and group size and work experience. The observed effect sizes, expressed as partial eta squared (ηp²), were small to moderate, suggesting that the associations between time, group size, and professional experience explained only a modest proportion of the variance in safety attitudes.

RoH simulations have been increasingly implemented in healthcare and nursing education [18, 19, 24]. However, RoH simulations conducted specifically in nursing homes with longitudinal evaluations are still lacking.

Well-educated and experienced staff members are more professional and show more responsibility for patient safety [25]. They can better understand changes in a patient’s state of health and are more aware of quality issues [12]. The study findings of [12] revealed negative attitudes for all safety domains. In contrast, Daupin et al. (2016) showed that most participants intended to change their practices following their exposure to the simulation [10], which suggests a positive attitude towards mistakes. In our study, all the nurses demonstrated a high patient safety attitude overall.

The present study showed that professional experience was associated with differences in attitudes toward the subdomain “working hours,” which is conceptually linked to error susceptibility. However, the study did not assess actual error rates. Kiesewetter et al. (2017) also identified working hours as a risk for errors [22].

In addition, an interaction effect between time and group size was found for the subdomain ‘importance of patient safety in the curriculum’ (*p* = .033). Regarding working hours and susceptibility to errors, the nurses with more professional experience (4th quartile) showed higher values post-evaluation, followed by a decrease at the three-month follow-up. In contrast, participants with less experience showed a slight but consistent increase over time. From a clinical perspective, these findings suggest that both group composition and professional experience may influence how staff engage with patient safety topics in training sessions. Smaller or more homogeneous groups may initially benefit more from RoH interventions, and experienced staff may show stronger immediate learning effects. However, without ongoing reinforcement, these effects tend to fade. This underlines the importance of integrating RoH simulations into a continuous learning strategy rather than offering them as one-time events. Furthermore, attitudes alone may not be sufficient to create sustained safety improvements; broader organizational efforts and leadership engagement are likely needed to maintain long-term culture change.

Lee et al. (2023) emphasized the need for further research concerning the influence of group size in RoH simulations [19]. In the present study, the smaller groups demonstrated higher patient safety attitude scores immediately after the intervention than the larger groups. However, after three months, the scores of both the small and large groups converged. In our study, the effect of group size was observed only for the subdomain “importance of patient safety in the curriculum.” This suggests that smaller groups may have a short-term benefit in fostering positive attitudes toward the importance of patient safety education. Beyond our study, prior research on simulation-based learning [26] has emphasized that smaller group sizes can generally promote greater engagement and reflective learning. However, the long-term retention of improved attitudes may require repeated interventions and broader integration into patient safety culture.

The present results imply that the RoH intervention is a promising low-threshold approach for enhancing nursing staff’s attitude toward patient safety in nursing homes. Integrating regular educational staff training could provide favorable opportunities for reflective learning and identify latent safety risks. For further RoH simulations, the present study suggests that smaller group sizes may be more favorable for improving attitudes toward the “importance of patient safety in the curriculum.” However, this effect was not observed across all subdomains of patient safety attitudes. In addition, since the overall attitude scores did not show a large or sustained effect after three months, implementing regular RoH trainings should be considered and evaluated with regard to both nurses’ attitudes and actual safety outcomes in nursing homes, in order to determine optimal training intervals.

## Limitations

This study has several limitations. First, the lack of a control group or a different intervention may limit the interpretation of the present results. A significant limitation of this study is the high drop-out rate after three months, which may affect the representativeness and interpretability of follow-up results. In addition, the G-APSQ-short may not be the most appropriate instrument to evaluate the effects of the RoH intervention, as it may not fully capture situational learning outcomes or behavioral changes resulting from simulation-based formats.These limitations may limit the generalizability of the present results.

## Conclusion

The present study highlights the potential of low-fidelity RoH simulation to support the development of patient safety awareness among staff of nursing homes. RoH simulations offer a feasible, scalable, and well-appreciated approach to enhance nurses’ attitudes toward patient safety. However, sustained improvements in safety attitudes will likely need multifaceted, repeated educational interventions embedded within a supportive organizational culture. Future studies should seek to replicate this study approach in other long-term care settings and evaluate its effects on nurses’ attitudes and patient safety outcomes.

## Data Availability

The datasets used and analyzed during the current study are available from the corresponding author upon reasonable request.

## Abbreviations

G-APSQ-short: German version of the Attitudes Towards Patient Safety Questionnaire (short form)
GLM: Generalized Linear Model
ICN: International Council of Nurses
RoH: Room of Horrors
WHO: World Health Organization

## References

[1] WHO. Patient safety incident reporting and learning systems: technical report and guidance. 2020. Available from: https://iris.who.int/handle/10665/334323.

[2] Slawomirski L KN. The economics of patient safety: from analysis to action. OECD health working papers. 2022;145:1–74.

[3] Hodkinson A, Tyler N, Ashcroft DM, Keers RN, Khan K, Phipps D, et al. Preventable medication harm across health care settings: a systematic review and meta-analysis. BMC Med. 2020;18(1):313. 33153451 10.1186/s12916-020-01774-9 PMC7646069

[4] Panagioti M, Khan K, Keers RN, Abuzour A, Phipps D, Kontopantelis E, et al. Prevalence, severity, and nature of preventable patient harm across medical care settings: systematic review and meta-analysis. BMJ. 2019;366:l4185. 31315828 10.1136/bmj.l4185 PMC6939648

[5] Kiljunen O, Savela R-M, Välimäki T, Kankkunen P. Managers’ perceptions of the factors affecting resident and patient safety work in residential settings and nursing homes: a qualitative systematic review. Res Nurs Health. 2024;47(4):397–408. 38522016 10.1002/nur.22382

[6] Rantz M, Ersek M, Care, Delivery. Quality Measurement, and quality improvement in nursing homes: issues and recommendations from the National academies’ report on the quality of care in nursing homes. J Am Geriatr Soc. 2023;71(2):329–34. 36795629 10.1111/jgs.18275

[7] Aase I, Ree E, Johannessen T, Strømme T, Ullebust B, Holen-Rabbersvik E, et al. Talking about quality: how ‘quality’ is conceptualized in nursing homes and homecare. BMC Health Serv Res. 2021;21(1):104. 33516206 10.1186/s12913-021-06104-0 PMC7847031

[8] Keers RN, Williams SD, Cooke J, Ashcroft DM. Causes of medication administration errors in hospitals: a systematic review of quantitative and qualitative evidence. Drug Saf. 2013;36(11):1045–67. 23975331 10.1007/s40264-013-0090-2 PMC3824584

[9] de Bienassis K, Llena-Nozal A, Klazinga NS. The economics of patient safety Part III: Long-term care valuing safety for the long haul. OECD Health Working Papers. 2021(121).

[10] Daupin J, Atkinson S, Bedard P, Pelchat V, Lebel D, Bussieres JF. Medication errors room: a simulation to assess the medical, nursing and pharmacy staffs’ ability to identify errors related to the medication-use system. J Eval Clin Pract. 2016;22(6):907–16. 27184006 10.1111/jep.12558

[11] Pickens J. Attitudes and perceptions. In: Borkowski N, Jones and Bartlett, editors. Organizational behavior in health care. Sudbury, ON, Canada; 2005.

[12] Alzahrani N, Jones R, Abdel-Latif ME. Attitudes of doctors and nurses toward patient safety within emergency departments of a Saudi Arabian hospital: a qualitative study. BMC Health Serv Res. 2019;18. 10.1186/s12913-018-3542-7 PMC6156948 30253774

[13] Al-Mugheed K, Bayraktar N, Al-Bsheish M, AlSyouf A, Jarrar M, AlBaker W et al. Patient safety attitudes among doctors and nurses: associations with workload, adverse events, experience. Healthcare (Basel). 2022;10(4). 10.3390/healthcare10040631 PMC9025351 35455809

[14] Kim J, Lee M, Hong E. Evaluating the outcomes of patient safety education programs in nursing education: a scoping review. BMC Nurs. 2025;24(1):273. 40075344 10.1186/s12912-025-02858-8 PMC11900140

[15] Lee SE, Morse BL, Kim NW. Patient safety educational interventions: A systematic review with recommendations for nurse educators. Nurs Open. 2022;9(4):1967–79. 34047058 10.1002/nop2.955 PMC9190690

[16] Niederhauser AG, Schwappach K. D. Interaktives Lernen im Room of Horrors. Manual für alters- und pflegeheime [Interactive learning in Room of Horrors. A manual for nursing homes]. 2021.

[17] Wiest K, Farnan J, Byrne E, Matern L, Cappaert M, Hirsch K, et al. Use of simulation to assess incoming interns’ recognition of opportunities to choose wisely. J Hosp Med. 2017;12(7):493–7. 28699935 10.12788/jhm.2761

[18] Zimmermann C, Fridrich A, Schwappach DLB. Training situational awareness for patient safety in a room of horrors: an evaluation of a Low-Fidelity simulation method. J Patient Saf. 2021;17(8):e1026–33. 33395018 10.1097/PTS.0000000000000806 PMC8612898

[19] Lee SE, Repsha C, Seo WJ, Lee SH, Dahinten VS. Room of horrors simulation in healthcare education: a systematic review. Nurse Educ Today. 2023;126:105824. 37121075 10.1016/j.nedt.2023.105824

[20] WHO. Conceptual framework for the international classification for patient safety. final technical report. 2009.

[21] Zigmont JJ, Kappus LJ, Sudikoff SN. The 3D model of debriefing: defusing, discovering, and deepening. Semin Perinatol. 2011;35(2):52–8. 21440811 10.1053/j.semperi.2011.01.003

[22] Kiesewetter J, Kager M, Fischer MR, Kiesewetter I. Validation of a German short version of the attitudes towards patient safety questionnaire (G-APSQsort) for the measurement of undergraduate medial students’ attitudes to and needs for patient safety. GMS J Med Educ. 2017;41(1):Doc8. 10.3205/zma001085 PMC5327660 28293675

[23] Kaveh O, Sharif-Nia H, Hosseini Z, Kaur H, Shafipour V. Psychometrics evaluation of the Persian version of attitudes toward patient safety questionnaire (APSQ-III) in nursing students. BMC Med Educ. 2024;24(1):1419. 39633360 10.1186/s12909-024-06318-w PMC11619130

[24] Löber N, Garske C, Rohe J. Room of horrors – ein low-fidelity simulationstraining für patientensicherheitsrelevante Gefährdungspotentiale Im klinikalltag [Room of horrors: a low-fidelity simulation practice for patient safety-relevant hazards of hospitalization]. Z Evid Fortbild Qual Gesundhwes. 2020; 153–154: 104–10. 32712178 10.1016/j.zefq.2020.05.010

[25] Al Malki A, Endacott R, Innes K. Health professional perspectives of patient safety issues in intensive care units in Saudi Arabia. J Nurs Manag. 2018;26(2):209–18. 28960563 10.1111/jonm.12536

[26] Lee J-Y, Lee SH, Kim J-H. A review of the curriculum development process of simulation-based educational intervention studies in Korea. Nurse Educ Today. 2018;64:42–8. 29459191 10.1016/j.nedt.2018.01.029

[27] Borde T, Satzung der Ethikkommission der Alice Salomon Hochschule Berlin [Regulations Governing the Ethics Committee of Alice Salomon University of Applied Sciences Berlin]. Ethic Commitee; 2019 [2019 Nov 06]. Available from: https://www.ash-berlin.eu/fileadmin/Daten/Forschung/2_Formulare/Satzung_Ethikkommission.pdf.

